# New Appliances and Things Medical

**Published:** 1901-12-14

**Authors:** 


### NEW APPLIANCES AND THINGS MEDICAL.

### VAN HOUTEN'S SOLUBLE COCOA.

(AGENT: ALEXANDER LECKIE, TOWER BUILDINGS, 33 TRINITY SQUARE, LONDON, E.C.)

THIS cocoa, as is well known, was practically the first example of a pure soluble preparation. Since its appearance in the market, the fashion has been followed by many other manufacturers, but among cocoas of this kind Van Houten's still retains the premier position for purity and excellence of flavour. One of the chief advantages of a cocoa which is soluble is, that the fat which it contains and which constitutes one of its most valuable food elements, is presented in a form which is well tolerated by the most delicate stomach, owing to the absence of any material excess of free fat. Van Houten's cocoa is strongly to be recommended for invalid use and for the nursery.

